# Lithium vanado(V)molybdate(VI), Li[VMoO_6_]

**DOI:** 10.1107/S1600536813022411

**Published:** 2013-08-17

**Authors:** Safa Ezzine Yahmed, Rawia Nasri, Mohamed Faouzi Zid, Ahmed Driss

**Affiliations:** a Laboratoire de Matériaux et Cristallochimie, Faculté des Sciences de Tunis, Université de Tunis ElManar, 2092 Manar II Tunis, Tunisia

## Abstract

Brannerite-type Li[VMoO_6_] has been synthesized by a solid state reaction route. The V and Mo atoms statistically occupy the same site with mirror symmetry and are octa­hedrally surrounded by O atoms. The framework is two-dimensional and is built up from edge-sharing (V,Mo)O_6_ octa­hedra forming (VMoO_6_)_∞_ layers that run parallel to the (001) plane. Li^+^ ions are situated in position with symmetry 2/*m* in the inter­layer space. The bond-valence analysis reveals that the Li^+^ ionic conductivity is along the [010] and [110] directions, and shows that this material may have inter­esting conduction properties. This simulation proposes a model of the lithium conduction pathways.

## Related literature

For background to lithium-ion batteries, see: Delmas *et al.* (1999 ▶); Cabana *et al.* (2003 ▶); Yin *et al.* (2003 ▶); Morgan *et al.* (2002 ▶); Gaubicher *et al.* (2000 ▶); Sato *et al.* (2000 ▶); Fu *et al.* (2010 ▶); Mikhailova *et al.* (2010 ▶). For details of structural relationships with other compounds, see: Andersson & Magnéli (1950 ▶); Darriet & Galy (1968 ▶); Karen *et al.* (2006 ▶); Knyazev *et al.* (2009 ▶); Mocala & Ziolkowski (1987 ▶); Mucha *et al.* (1999 ▶); Ruh & Wadsley (1966 ▶); Shklover *et al.* (1996 ▶). For details of the bond-valence method, see: Brown, (2002 ▶). For BVS pathway simulation, see: Mazza *et al.* (2002 ▶); Ben Smida *et al.* (2013 ▶); Mazza (2001 ▶); Ouerfelli *et al.* (2007*a* ▶,*b* ▶).

## Experimental

### 

#### Crystal data

LiMoVO_6_
*M* *_r_* = 249.82
Monoclinic, 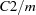

*a* = 9.3555 (9) Å
*b* = 3.6432 (5) Å
*c* = 6.6887 (7) Å
β = 111.669 (6)°
*V* = 211.87 (4) Å^3^
*Z* = 2
Mo *K*α radiation
μ = 5.10 mm^−1^
*T* = 298 K
0.29 × 0.22 × 0.14 mm

#### Data collection

Enraf–Nonius CAD4 diffractometer
Absorption correction: ψ scan (North *et al.*, 1968 ▶) *T*_min_ = 0.274, *T*_max_ = 0.498
876 measured reflections
354 independent reflections
350 reflections with *I* > 2σ(*I*)
*R*_int_ = 0.018
2 standard reflections every 120 min intensity decay: 1.1%

#### Refinement

*R*[*F* ^2^ > 2σ(*F* ^2^)] = 0.018
*wR*(*F* ^2^) = 0.052
*S* = 1.16
354 reflections
30 parameters
Δρ_max_ = 0.90 e Å^−3^
Δρ_min_ = −0.57 e Å^−3^

### 

Data collection: *CAD-4 EXPRESS* (Duisenberg, 1992 ▶; Macíček & Yordanov, 1992 ▶); cell refinement: *CAD-4 EXPRESS*; data reduction: *XCAD4* (Harms & Wocadlo, 1995 ▶); program(s) used to solve structure: *SHELXS97* (Sheldrick, 2008 ▶); program(s) used to refine structure: *SHELXL97* (Sheldrick, 2008 ▶); molecular graphics: *DIAMOND* (Brandenburg, 1998 ▶); software used to prepare material for publication: *WinGX* (Farrugia, 2012 ▶).

## Supplementary Material

Crystal structure: contains datablock(s) I. DOI: 10.1107/S1600536813022411/ru2052sup1.cif

Structure factors: contains datablock(s) I. DOI: 10.1107/S1600536813022411/ru2052Isup2.hkl

Additional supplementary materials:  crystallographic information; 3D view; checkCIF report

## Figures and Tables

**Fig. 1.**
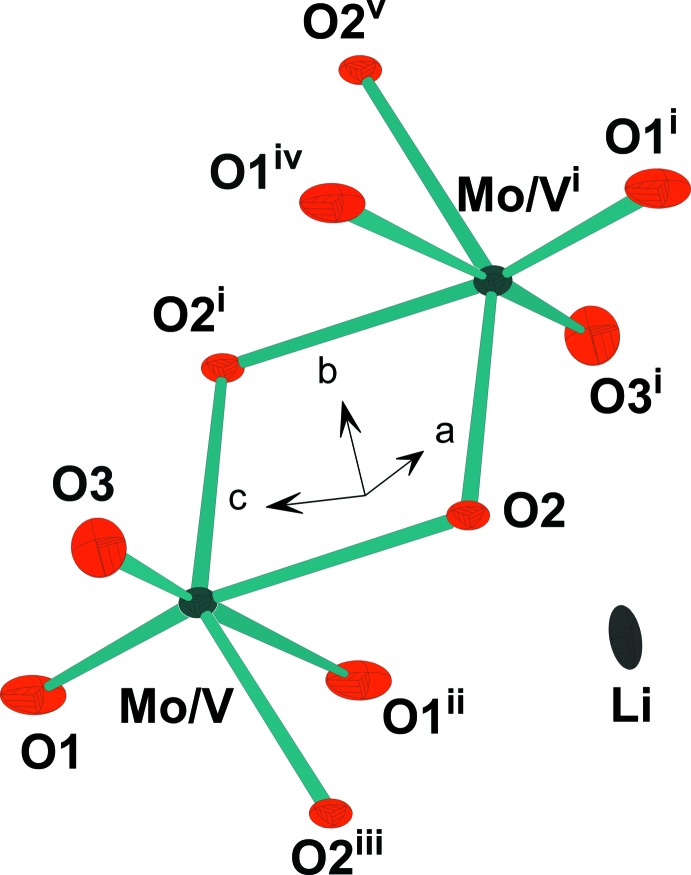
Unité asymétrique du composé LiMoVO6. Les éllipsoïdes d'agitation thermique ont été définis avec 50% de probabilité. [Codes de symétrie: (i) 1/2 - x, 1/2 + y, 1 - z; (ii) -x, y, 1 - z; (iii) 1/2 - x, -1/2 + y, 1 - z; (iv) 1/2 + x, 1/2 + y, z; (v) x, 1 + y, z.]

**Fig. 2.**
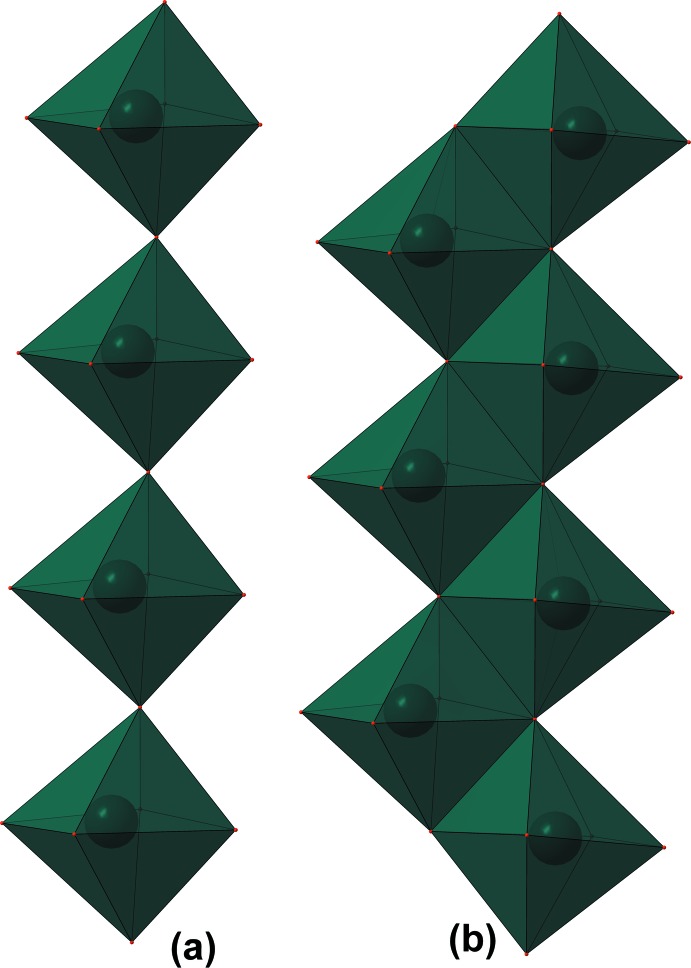
(a) Représentation d'une chaîne simple formée par les octaèdres MO6 (M=Mo/V). (b) Représentation d'une chaîne double.

**Fig. 3.**
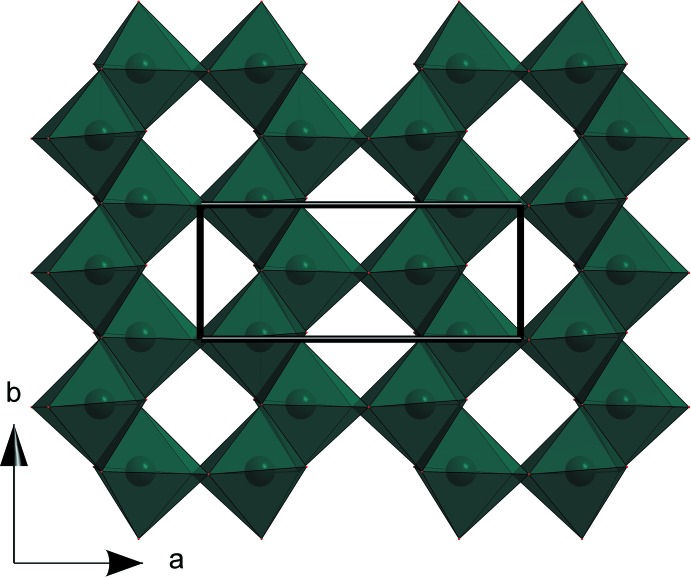
Projection d'une couche (VMoO6) selon c dans LiMoVO6.

**Fig. 4.**
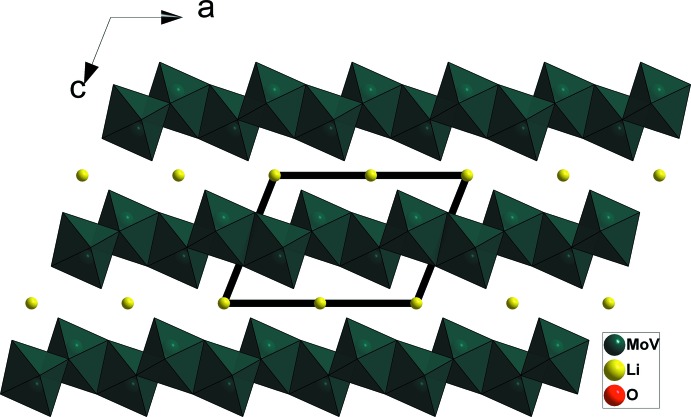
Projection de la structure du compsé LiMoVO6 selon b.

**Fig. 5.**
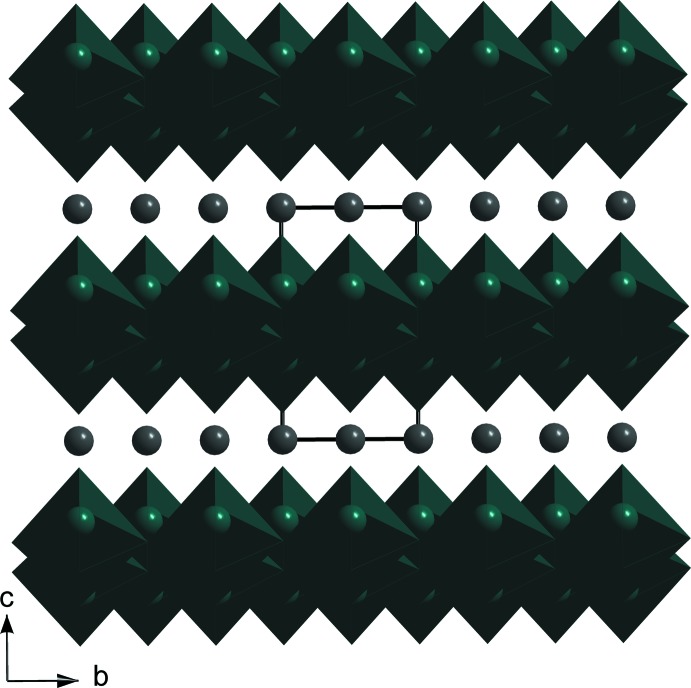
Projection de la structure du compsé LiMoVO6 selon a.

**Fig. 6.**
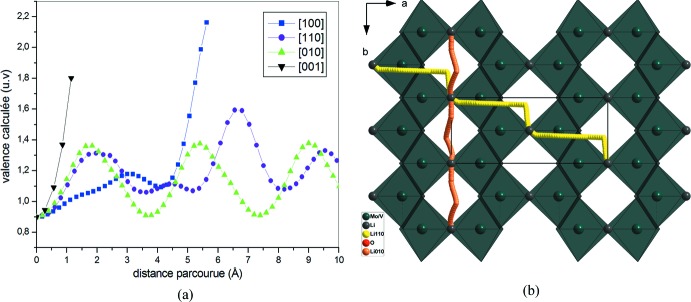
(a) Variation de la valence en fonction de la distance parcourue à partir de la position initiale de Li dans LiMoVO6. (b) Modélisation des chemins de conduction des cations Li+ selon les directions [010] et [110].

## supplementary crystallographic information

### 1. Comment

Les batteries Lithium-ion sont considérées comme source d'énergie intéressante, en raison de leurs densités d'énergie plus élevées par rapport aux autres systèmes de batteries rechargeables (Delmas *et al.*, 1999). Dans le but d'améliorer les proprriétés éléctrochimiques de la cathode dans ce type de batteries, plusieurs travaux antérieurs ont été effectués, (Cabana *et al.*, 2003; Yin *et al.*, 2003; Morgan *et al.*, 2002; Gaubicher *et al.*, 2000; Sato *et al.*, 2000; Fu *et al.*, 2010; Mikhailova *et al.*, 2010). En particulier, les matériaux à base de lithium et vanadium ont été largement étudiés vu leurs structures ouvertes, possédant des canaux (structure tridimensionnelle) ou des espaces intercouches (structure bidimensionnelle) suffisant pour une bonne mobilité de l'ion Li^+^ et en raison de la capacité du vanadium d'adopter plusieurs degrés d'oxydation. Ces matériaux présentent des performances éléctriques intéressantes en relation avec leurs structures.

Dans ce cadre, l'exploration du système Li–V–Mo–O nous a conduit à la phase monocristalline de formulation LiMoVO_6_ qui a été synthétisée par réaction à l'état solide.

La résolution de la structure révèle que les atomes de vanadium et de molybdène occupent le même site (position *sp*éciale 4i). *L*'unité *asym*étrique du composé étudié LiMoVO_6_ renferme deux octaèdres *M*O_6_ (*M* = Mo/V) liés par une arête pour former l'unité classique *M*_2_O_9_ (Fig. 1).

La structure, du vanadomolybdate synthétisé, peut être décrite à partir de la jonction de deux octaèdres *M*O_6_ (*M* = Mo/V) partageant des sommets et formant les groupements *M*_2_O_11_. Ces derniers se lient entre eux par ponts mixtes *M*—O—*M* pour former des chaînes simples classiques (*M*_2_O_9_)_∞_ (*M* = Mo/V) se propageant selon *b* (Fig. 2a). *L*'association par partage d'arêtes de celles-ci mène à des chaînes doubles (*M*_4_O_21_)_∞_ (Fig. 2 b) en zigzag qui s'étendent selon la direction [010]. La liaison de part et d'autre, entre ces dernières par pont O–*M*–O le long du plan *ac* (Fig. 3), conduit à des couches ondulées MoVO_6_ qui se présentent en dents de scies (Fig. 4). Il en résulte une structure bidimensionnelle dans laquelle les cation Li^+^ sont situés dans les espaces intercouches (Fig. 5).

La recherche d'une corrélation entre les caractéristiques structurales de notre matériau LiMoVO_6_ et ses propriétés physiques notamment de conductivité ionique, nous a encouragés à faire l'étude des chemins les plus probables de migration des cations Li^+^ par le modèle de calcul des valences (BVS) (Brown, 2002) qui a été appliqué par plusieurs auteurs notamment: pour la conduction ionique du lithium dans La_2/3-_*_x_*Li_3x_TiO_3_ (Mazza *et al.*, 2002) et LiCo_2_As_3_O_10_ (Ben Smida *et al.*, 2013), dans les structures Nasicon (Mazza, 2001) et dans les diarséniates TlFe_0.22_Al_0.78_As_2_O_7_ (Ouerfelli *et al.*, 2007*a*) et KFeAs_2_O_7_ (Ouerfelli *et al.*, 2007*b*): Le chemin de conduction dans un solide cristallin, peut être modélisé en calculant la somme des valences V_i_(*x*,*y*,*z*) dans une grille de points à l'intérieur du réseau anionique selon une direction initiale de migration, à partir de la position cristallographique du cation, déterminée depuis la diffraction des rayons *X*. Les points dans cette grille correspondant à la valence idéale de l'ion sont des positions énergétiquement favorisées, ainsi une valeur plus élevée représentant une cavité trop petite, donc avec une énergie potentielle répulsive plus élevée. La somme des valences V_i_(*x*,*y*,*z*) pour le cation mobile peut être tracée en fonction de la distance parcourue (*d*), obtenant ainsi la variation de la valence calculée en fonction de la distance parcourue d(Å) pour les différentes directions de migration (Fig. 6a).

Ainsi, les chemins de déplacement les plus probables, dans le cas de notre matériau, sont ceux selon les directions [010] et [110]. En effet, les valences maximales calculées du lithium selon l'axe *b* sont proches de 1,4 u.v (unité de valence) pour une distance dépassant 10 Å (Fig. 6a) alors qu'elles atteignent 1,6 u.v selon [110] (Fig. 6a). Le mouvement de migration des cations Li^+^ semble plus favorable selon l'axe *b*. De plus, la longueur de saut d'un site cristallographique de litium au prochain voisin est minimale selon *b* (3,643 Å selon [010] et 5,02 Å selon [110]). Donc, le mouvement d'ions Li^+^ le long de [110] semble être moins favorable. Quand aux directions [100] et [001], les valences calculées sont élevées (V_max_≈ 2 u.v) pour des distances courtes: Le lithium est presque Figé dans son site (V=1.8uv pour d= 1.1 Å) selon *c* alors qu'il rencontre des barrières énergétiques pour des distances parcourues courtes selon *a* (v=2.16 u.v pour d=5.6 Å). Les chemins de migrations selon les directions [010] et [110] sont matérialisés dans la figure (Fig. 6 b). La synthèse de la phase pure va nous permettre d'effectuer une étude de la conductivité ionique par spectroscopie d'impédance complexe.

Un balayage bibliographique montre que le composé étudié est isotype à certains vanadates de métaux bivalents *Me*V_2_O_6_ (*Me* = Mg, Mn, Co, Zn, Cu et Cd) (Mocala & Ziolkowski, 1987) ainsi que les matériaux NaVMoO_6_ (Darriet & Galy, 1968), LiVWO_6_ et NaVWO_6_ (Knyazev *et al.*, 2009). Ces vanadates ont tous une structure type Brannerite ThTiO_6_ (Ruh & Wadsley, 1966).

La comparaison de ce matériau avec le composé de formulation analogue KVMoO_6_ (Mucha *et al.*, 1999) montre une différence nette. En effet, les facteurs géométriques ainsi que la dimensionalité du matériau ont changés en raison de l'augmentation du rayon ionique du cation alcalin. La structure du matériau au potassium est unidimensionnelle et se présente sous forme de chaînes doubles (*M*_4_O_21_)_∞_ parallèles à l'axe *b*.

Le composé Ca_2_VMoO_6_(Karen *et al.*, 2006) est caractérisé par les chaînes simples (*M*_2_O_9_)_∞_ (*M* = Mo/V) rencontrées dans notre matériau. Elles se lient par mise en commun de sommets pour former des couches infinies qui se joignent par ponts mixtes *M*—O—*M* conduisant à une structure perovskite tridimensionnelle où les cations bivalents Ca^2+^ logent dans les canaux. Les atomes de molybdène et de vanadium, dans le matériau Ca_2_VMoO_6_, occupent partiellement les mêmes sites (position 4 b).

L'examin des phases V_2_O_5_ (Shklover *et al.*, 1996) et MoO_3_ (Andersson & Magnéli, 1950) révèle la présence des chaînes doubles formées par partage d'arêtes entre les polyèdres de vanadium (respectivement de molybdène) semblables à celles rencontrées dans notre matériau. Néanmoins, dans ces deux oxydes, les chaînes se lient par mise en commun de sommets pour former des charpentes bidimensionnelles.

### 2. Experimental

La phase LiMoVO_6_ a été obtenue par réaction à l'état solide à partir des réactifs: Li_2_CO_3_ (AZIENDA CHIMICA, 104094819), V_2_O_5_ (ACROS ORGANICS, A018448201) et (NH_4_)_2_Mo_4_O_13_ (Fluka, 69858) pris dans le rapport molaire Li:V:Mo = 1:3:1. Le mélange, finement broyé, est chauffé à 623 K pendant 12 h en vue d'éliminer NH_3_ et CO_2_. Après un broyage fin, le résidu final est placé dans une nacelle en porcelaine puis porté à 873 K, température proche de la fusion. Après trois jours, on commence un refroidissement lent par palier de 5 degrés puis rapide jusqu'à la température ambiante. Des cristaux de couleur jaunâtre sous forme de baguettes ont été isolés.

### 3. Refinement

Les densités d'électrons maximum et minimum restants dans la Fourier-différence sont acceptables et sont situées respectivements à 0,83 Å de V et à 0,80 Å de V.

### Crystal data

**Table:**

LiMoVO_6_	*F*(000) = 232
*M**_r_* = 249.82	*D*_x_ = 3.916 Mg m^−^^3^
Monoclinic, *C*2/*m*	Mo *K*α radiation, λ = 0.71073 Å
Hall symbol: -C 2y	Cell parameters from 25 reflections
*a* = 9.3555 (9) Å	θ = 11–16°
*b* = 3.6432 (5) Å	µ = 5.10 mm^−^^1^
*c* = 6.6887 (7) Å	*T* = 298 K
β = 111.669 (6)°	Prism, yellow
*V* = 211.87 (4) Å^3^	0.29 × 0.22 × 0.14 mm
*Z* = 2	

### Data collection

**Table:**

Enraf Nonius CAD4 diffractometer	350 reflections with *I* > 2σ(*I*)
Radiation source: fine-focus sealed tube	*R*_int_ = 0.018
Graphite monochromator	θ_max_ = 30.0°, θ_min_ = 3.3°
ω/2θ scans	*h* = −12→12
Absorption correction: ψ scan (North *et al.*, 1968)	*k* = −5→1
*T*_min_ = 0.274, *T*_max_ = 0.498	*l* = −9→9
876 measured reflections	2 standard reflections every 120 min
354 independent reflections	intensity decay: 1.1%

### Refinement

**Table:**

Refinement on *F*^2^	Primary atom site location: structure-invariant direct methods
Least-squares matrix: full	Secondary atom site location: difference Fourier map
*R*[*F*^2^ > 2σ(*F*^2^)] = 0.018	*w* = 1/[σ^2^(*F*_o_^2^) + (0.0273*P*)^2^ + 0.9791*P*] where *P* = (*F*_o_^2^ + 2*F*_c_^2^)/3
*wR*(*F*^2^) = 0.052	(Δ/σ)_max_ < 0.001
*S* = 1.16	Δρ_max_ = 0.90 e Å^−^^3^
354 reflections	Δρ_min_ = −0.57 e Å^−^^3^
30 parameters	Extinction correction: *SHELXL97* (Sheldrick, 2008), Fc^*^=kFc[1+0.001xFc^2^λ^3^/sin(2θ)]^-1/4^
0 restraints	Extinction coefficient: 0.008 (2)

### Special details

**Table:**

Geometry. All e.s.d.'s (except the e.s.d. in the dihedral angle between two l.s. planes) are estimated using the full covariance matrix. The cell e.s.d.'s are taken into account individually in the estimation of e.s.d.'s in distances, angles and torsion angles; correlations between e.s.d.'s in cell parameters are only used when they are defined by crystal symmetry. An approximate (isotropic) treatment of cell e.s.d.'s is used for estimating e.s.d.'s involving l.s. planes.
Refinement. Refinement of *F*^2^ against ALL reflections. The weighted *R*-factor *wR* and goodness of fit *S* are based on *F*^2^, conventional *R*-factors *R* are based on *F*, with *F* set to zero for negative *F*^2^. The threshold expression of *F*^2^ > σ(*F*^2^) is used only for calculating *R*-factors(gt) *etc*. and is not relevant to the choice of reflections for refinement. *R*-factors based on *F*^2^ are statistically about twice as large as those based on *F*, and *R*-factors based on ALL data will be even larger.

### Fractional atomic coordinates and isotropic or equivalent isotropic displacement parameters (Å^2^)

**Table:**

	*x*	*y*	*z*	*U*_iso_*/*U*_eq_	Occ. (<1)
Mo	0.18622 (4)	0.0000	0.65068 (5)	0.00752 (17)	0.50
V	0.18622 (4)	0.0000	0.65068 (5)	0.00752 (17)	0.50
Li	0.0000	0.0000	0.0000	0.0152 (17)	
O1	0.0259 (3)	0.0000	0.7107 (5)	0.0167 (5)	
O2	0.3080 (3)	0.0000	0.4362 (4)	0.0096 (4)	
O3	0.3319 (3)	0.0000	0.8860 (4)	0.0173 (5)	

### Atomic displacement parameters (Å^2^)

**Table:**

	*U*^11^	*U*^22^	*U*^33^	*U*^12^	*U*^13^	*U*^23^
Mo	0.0082 (2)	0.0049 (2)	0.0100 (2)	0.000	0.00406 (14)	0.000
V	0.0082 (2)	0.0049 (2)	0.0100 (2)	0.000	0.00406 (14)	0.000
Li	0.015 (4)	0.026 (5)	0.006 (3)	0.000	0.005 (3)	0.000
O1	0.0108 (10)	0.0092 (12)	0.0320 (14)	0.000	0.0102 (10)	0.000
O2	0.0118 (10)	0.0039 (10)	0.0159 (10)	0.000	0.0083 (8)	0.000
O3	0.0131 (11)	0.0221 (15)	0.0151 (11)	0.000	0.0032 (9)	0.000

### Geometric parameters (Å, º)

**Table:**

Mo—O3	1.659 (3)	Li—O3^vi^	2.3423 (16)
Mo—O1	1.690 (3)	Li—O3^i^	2.3423 (16)
Mo—O2^i^	1.9189 (8)	O1—Li^vii^	2.037 (3)
Mo—O2^ii^	1.9189 (8)	O1—Mo^iii^	2.497 (3)
Mo—O2	2.136 (2)	O2—V^i^	1.9189 (8)
Mo—O1^iii^	2.497 (3)	O2—Mo^i^	1.9189 (8)
Li—O1^iv^	2.037 (3)	O2—V^ii^	1.9189 (8)
Li—O1^iii^	2.037 (3)	O2—Mo^ii^	1.9189 (8)
Li—O3^ii^	2.3423 (16)	O3—Li^viii^	2.3423 (16)
Li—O3^v^	2.3423 (16)	O3—Li^ix^	2.3423 (16)
			
O3—Mo—O1	105.39 (14)	O3^ii^—Li—O3^vi^	77.90 (10)
O3—Mo—O2^i^	100.50 (8)	O3^v^—Li—O3^vi^	102.10 (10)
O1—Mo—O2^i^	101.46 (7)	O1^iv^—Li—O3^i^	90.48 (8)
O3—Mo—O2^ii^	100.50 (8)	O1^iii^—Li—O3^i^	89.52 (8)
O1—Mo—O2^ii^	101.46 (7)	O3^ii^—Li—O3^i^	102.10 (10)
O2^i^—Mo—O2^ii^	143.35 (14)	O3^v^—Li—O3^i^	77.90 (10)
O3—Mo—O2	100.49 (11)	O3^vi^—Li—O3^i^	180.00 (14)
O1—Mo—O2	154.13 (13)	Mo—O1—Li^vii^	130.75 (16)
O2^i^—Mo—O2	73.43 (7)	Mo—O1—Mo^iii^	103.17 (14)
O2^ii^—Mo—O2	73.43 (7)	Li^vii^—O1—Mo^iii^	126.08 (11)
O3—Mo—O1^iii^	177.79 (11)	V^i^—O2—Mo^i^	0.00 (2)
O1—Mo—O1^iii^	76.83 (14)	V^i^—O2—V^ii^	143.35 (14)
O2^i^—Mo—O1^iii^	78.93 (8)	Mo^i^—O2—V^ii^	143.35 (14)
O2^ii^—Mo—O1^iii^	78.93 (8)	V^i^—O2—Mo^ii^	143.35 (14)
O2—Mo—O1^iii^	77.30 (9)	Mo^i^—O2—Mo^ii^	143.35 (14)
O1^iv^—Li—O1^iii^	180.00 (14)	V^ii^—O2—Mo^ii^	0.00 (2)
O1^iv^—Li—O3^ii^	90.48 (8)	V^i^—O2—Mo	106.57 (7)
O1^iii^—Li—O3^ii^	89.52 (8)	Mo^i^—O2—Mo	106.57 (7)
O1^iv^—Li—O3^v^	89.52 (8)	V^ii^—O2—Mo	106.57 (7)
O1^iii^—Li—O3^v^	90.48 (8)	Mo^ii^—O2—Mo	106.57 (7)
O3^ii^—Li—O3^v^	180.00 (14)	Mo—O3—Li^viii^	121.78 (8)
O1^iv^—Li—O3^vi^	89.52 (8)	Mo—O3—Li^ix^	121.78 (8)
O1^iii^—Li—O3^vi^	90.48 (8)	Li^viii^—O3—Li^ix^	102.10 (10)

Symmetry codes: (i) −*x*+1/2, −*y*−1/2, −*z*+1; (ii) −*x*+1/2, −*y*+1/2, −*z*+1; (iii) −*x*, −*y*, −*z*+1; (iv) *x*, *y*, *z*−1; (v) *x*−1/2, *y*−1/2, *z*−1; (vi) *x*−1/2, *y*+1/2, *z*−1; (vii) *x*, *y*, *z*+1; (viii) *x*+1/2, *y*+1/2, *z*+1; (ix) *x*+1/2, *y*−1/2, *z*+1.
